# Local resource mobilization for malaria vector control among Rwandan rice farmers: a pilot study into the role of community ownership

**DOI:** 10.1186/s41043-023-00345-x

**Published:** 2023-01-23

**Authors:** Alexis Rulisa, Luuk van Kempen, Emmanuel Hakizimana, Constantianus J. M. Koenraadt

**Affiliations:** 1 Department of Cultural Anthropology and Development Studies, and Radboud Social Cultural Research, Radboud University, 6525 XZ Nijmegen, The Netherlands; 2 Malaria and Other Parasitic Diseases Division, Rwanda Biomedical Center, 7162 Kigali, Rwanda; 3 Laboratory of Entomology, Wageningen University & Research, 6708 Wageningen, The Netherlands

**Keywords:** Localisation, Community ownership, Malaria control, Larval source management, Rice, Willingness-to-pay, Rwanda

## Abstract

**Background:**

Evidence suggests a vicious cycle between rice cultivation and malaria control in Rwanda. Rice fields offer an attractive breeding ground for malaria vectors, which increases the disease burden in rice farming communities, and, consequently, reduces productivity in the rice sector. Community-based larval source management in rice fields is propagated as a sustainable solution to break this cycle. A sense of agency and ownership of malaria control interventions, as well as the mobilization of resources at the local level, are often considered preconditions for success. However, an evidence gap exists regarding the interaction between the agentive and financial dimension of local sustainability.

**Methods:**

We conduct a larviciding pilot involving three groups; one group where rice farmers sprayed their fields under expert supervision, one group where rice farmers organised the larviciding campaign themselves, and a (non-sprayed) control group. We test whether the difference in agency between the intervention groups affects farmers’ willingness-to-pay for a larviciding campaign. Willingness-to-pay is elicited in a contingent valuation exercise, more specifically a bidding game, and is assessed both before and after the pilot (*n* = 288). Difference-in-difference estimates are computed, using a propensity score matching technique. Supplementary data were collected in a survey and two focus group discussions for triangulation.

**Results:**

The high-agency (self-organised) group significantly outperforms the low-agency (expert-supervised) group in terms of maintaining its willingness to contribute financially. However, higher willingness-to-pay in the high-agency group does not appear to be driven by a stronger sense of ownership per se. The supplementary data indicate high levels of ownership in both treatment groups compared to the control group. A tentative explanation lies in diverging perceptions concerning the effectiveness of the pilot.

**Conclusions:**

The study supports the idea that community-led organization of larval source management can prove instrumental in mobilizing finance for malaria control in low-income settings where rice production interferes with the fight against malaria. However, the causality is complex. Feelings of ownership do not appear the main driver of willingness-to-pay, at least not directly, which opens up the possibility of initiating community-driven malaria control interventions that promote the agentive and financial dimension of local sustainability simultaneously.

## Background

While the Sustainable Development Goals (SDGs) are often identified by their global nature, a push for ‘localisation’ of the goals is ongoing, defined as “the process of defining, implementing and monitoring strategies at the local level for achieving global, national and subnational sustainable development goals and targets” (p. 6) [1]. Localisation has primarily directed the spotlight towards local government actors (see e.g. [2]), but in line with its original intention, the engagement of non-governmental local stakeholders, including communities themselves, is increasingly acknowledged as a key to success in ‘localizing sustainability’ [3]. This paper addresses the potential for localisation of a particular SDG sub-target, i.e., ending the malaria epidemic by 2030 (target 3.3; indicator 3.3.3), in the context of Rwanda, where malaria still represented the third most important cause of lost disability-adjusted life years (for all ages combined) in 2019 [4]. While the Rwandan government has mainly relied on the distribution of insecticide-treated nets (ITNs) and indoor residual spraying (IRS) campaigns to fight malaria transmission, it has started to deploy larval source management (LSM), in particular biological larviciding, more widely as a malaria prevention instrument since 2020. Concerns about insecticide resistance in African *Anopheles* ssp. mosquitoes [5] as well as evidence of increased outdoor biting [6] provide a strong rationale for LSM as a complement to indoor interventions. It has officially been added to the government’s malaria vector control mix in its Malaria Strategic Plan 2020–2024, in alignment with the World Health Organization’s recommendation to prioritise larviciding if mosquito breeding sites are ‘few, fixed, and findable’ [7]. Since it is a relatively novel tool in Rwanda, the sustainability of future LSM efforts warrants attention.

Already in the pre-SDG era calls were sounded to engage local communities in LSM in the context of Sub-Sahara Africa, based on pilots in Kenya and Tanzania [8]. However, larviciding interventions have remained strongly expert-driven in most cases, because larval control “requires unusual specialist skills”, including a “basic minimum of ecological understanding” (p. 2) [8]. More recently, under the impetus of the SDG localisation agenda and the rise of ‘citizen science’ in the health and biomedical field [9], larviciding projects with varying degrees of community involvement have proliferated across Sub-Saharan Africa. An evidence base is gradually emerging from evaluations of these initiatives, on which we purport to build by reporting on a larviciding pilot among rice-cultivating communities in Rwanda. Most evaluations that have appeared so far are either quantitative studies assessing the effectiveness of community-based larviciding in terms of reduced mosquito density and malaria risk, or, alternatively, qualitative inquiries into how communities have experienced their participation in the larviciding process, i.e., gauging the extent to which local ownership has been achieved. Our objective is a different yet related one, as we use both elements –effectiveness, at least in perception, and experiences of process ownership– to understand the communities’ willingness to co-finance LSM. Hence, this study explores the potential for local resource mobilization towards community-based LSM and thereby aims to shed more light on the financial sustainability dimension of routine LSM implementation.

The choice for rice-cultivation communities is motivated by systematic evidence from Sub-Saharan Africa that the expansion of irrigated rice fields is bound to drive up local malaria prevalence [10]. Malaria vector densities in rice-growing communities tend to be a multiple of those in non-rice-growing areas, leading to higher incidence, despite the fact that the surplus income from rice has some mitigating potential, e.g. in the form of more mosquito-proof housing. Such a negative externality from rice cultivation on public health is also witnessed to operate in our study area in Eastern Rwanda [11]. This problem is likely to persist in the coming years, as the government’s National Rice Development Strategy 2021–2030 (NRDS-II) envisages doubling the area available for rice cultivation through conversion and rehabilitation of marshlands.

For the larviciding pilot, we engaged with four rice cooperatives in the sub-district of Ruhuha, each representing farmers in a separate area of marshland. Members routinely make financial contributions to their cooperative for collective purposes, such as the purchase of pesticides and fertilizers, which renders cooperatives a suitable entry point for studying local willingness to allocate resources to LSM. Our identification strategy for assessing the role of local ownership in willingness-to-pay (WTP) is based on variation in the level of community engagement across sites; larviciding was expert-led in one site and community-led in two other sites. The fourth site served as control (non-intervention) site. We compare pre-intervention and post-intervention WTP across the treatment arms, where it should be noted that WTP is ‘stated’ rather than ‘revealed’. The larviciding pilot, including the community-led modality, was fully funded from external resources. Ex-post inquiries, both quantitative and qualitative, into perceptions of ownership and effectiveness of the campaign were carried out to establish links with the observed WTP dynamics.

Before introducing the methodology of the study and offering details on the research setting and study design, we first review the literature on local ownership and shed light on the existing empirical evidence base on community-oriented LSM initiatives in Sub-Saharan Africa to clarify our analytical framework and pinpoint the knowledge gap it addresses.

### Local ownership: agency versus commitment

Under different labels and guises, global development actors have since long embraced the ambition to ‘localise’ development work, often adopting a model akin to community-based or community-driven development [12]. Communities are typically conceived as villages, neighbourhoods, or social groups with a specific common interest, such as producer associations, that make collective decisions [13]. The intended benefit of this ‘local turn’ is commonly formulated in terms of enhanced ‘local ownership’, even though critical scholars have denounced the obfuscation around the actual interpretation of both the terms ‘local’ and ‘ownership’ [14, 15]. Recently, the ‘localisation agenda’ has received new impetus and gained wider currency among hitherto less involved parts of the global development community, such as in humanitarian action and peacebuilding [16], and philanthropy [17]. The ‘shift the power’ movement is one of its most prominent manifestations. Academic reflections on this renewed localisation drive prove helpful in understanding the meaning of local ownership and in recognizing where community co-financing of development solutions comes into the picture.

Baguios et al. [17] distinguish three dimensions of locally-led development, two of which have a direct link to our study; the ‘resources’ and ‘agency’ dimensions. The ‘resource’ aspect primarily entails a call for international actors to channel funds directly to (community-based) organizations in the Global South, which is different from community co-financing schemes. Yet, it indirectly includes such schemes by referring to the ‘community philanthropy’ concept, in which local resource mobilization is considered a key component, supporting self-reliance [18]. The second dimension of ‘agency’ rather focuses on self-governance and self-determination, as it aims to strengthen local actors’ ability to design and implement their own solutions. This distinction relates to competing interpretations of ‘local ownership’, as originally identified by Fraser and Whitfield [19]. Local co-financing is most aptly associated with fostering ‘ownership as *commitment*’, which translates as the willpower to pursue a particular solution (also into the future), regardless of how this solution came about. In this interpretation, ownership is a predictor of how sustainable a solution will be. By contrast, the agency dimension is more closely associated with ‘ownership as *control*’, in the sense of local actors having (autonomous) decision-making power regarding the process and outcomes of development solutions [17].

Ownership as commitment or control have different antecedents in development thinking. The ‘control’ (agency) element is rooted in a long tradition of ‘participatory development’ linked to empowerment objectives [20], whereas ownership as commitment is most prominent in the work of development economists studying local public goods provision. As an exponent of this public goods literature, Walker [21] explains that community fundraising acts as an ex-ante test of whether the concerned community actually values the solution at hand. In addition, co-payments give community members a stake in maintaining the public good that they value. This is most tangible in case of physical infrastructure, such as water and sanitation facilities, but should more broadly prevent unsustainable, and therefore wasteful, projects. Contributions in this line tend to stress the shift from an external dependency mindset to a spirit of self-reliance.

Returning to the global fight against malaria, let us consider the extent to which notions of local ownership have been integrated in malaria control efforts, and the relative importance of the agency/control and resource/commitment aspects. A first observation is that donors focusing on malaria tend to be latecomers to the ‘local turn’, as witnessed by Baltzell et al. [22]:

In light of stalled progress, flatlined funding, and the increasing complexity of identifying and treating malaria cases, the global community is looking to operational solutions to increase the effective delivery of interventions for malaria. The topic of community engagement is at the forefront of this conversation and is recognized as an essential component in the shift toward creating local and site-specific solutions. Community engagement strategies have long been incorporated into themes such as women’s health, political action, and HIV/AIDS.… [T]he malaria community has only recently begun to consider the significance and potential of community engagement for malaria elimination (p. 2).

A first scoping review of malaria prevention and control interventions that have incorporated some form of community engagement is still under way [23], but advocacy contributions provide some insight. For example, Whittaker and Smith [24] propagate community engagement (CE) for malaria elimination and distinguish the following four escalating degrees: (1) passive community acceptance of an intervention; (2) moderate participation in the form of community adherence to low-effort activities; (3) active participation in high-effort activities, and (4) community ownership where responsibilities are transferred to communities. The authors observe that agencies fighting malaria have tended to believe that reaching the first or second step of the participation ladder was a sufficient condition for success, while climbing the ladder towards community ownership (step 4) should be the aim, most urgently in the elimination phase (p. 2).

While the steps on the ladder are defined according to the community’s level of agency, the rationale behind the appeal for community ownership principally alludes to ownership as commitment rather than agency. Strong community engagement is considered “vital to ensure sustained political commitment and funding for malaria” (p. 4), and, consequently, programs “will be much more likely to transfer responsibility and costing for some ongoing activities to individuals and communities” (p. 5). Hence, the implicit assumption is that enhanced agency for communities opens up opportunities for community co-financing and thereby to more sustainable funding for malaria interventions. Put differently, ‘ownership as control’ strengthens ‘ownership as commitment’.

The idea that agency is a stepping stone towards (future) financial commitment seems to enjoy wider support, as we have not been able to identify programmes, at least in Sub-Saharan Africa, that introduce agency-oriented community engagement and local resource mobilization simultaneously, except for a programme in Nigeria that focuses on integrated case management of childhood illnesses, including malaria [25]. This is not to say that an explicit discussion on local resource mobilization for malaria control is absent, but ‘local ownership’ in these contributions refers to country ownership rather than community ownership. For example, it is argued that governments in the Global South should break out of ‘neo-dependency’ and procure bed-nets and antimalarials themselves rather than relying on procurement by international donors, but the implications of such a shift to the community level are not traced [26].

### Community-based larval source management

In this sub-section we highlight selected studies reporting on experiments in Sub-Saharan Africa where LSM interventions were conducted with community participation in some form. Seven projects have been identified, covering Botswana, Burkina Faso, Ethiopia, Kenya, Malawi, Tanzania, and Zimbabwe, all of which applied *Bacillus thuringiensis* var. *israelensis* (*Bti*). Table 1 provides basic profiles of these initiatives in terms of timing, scale, and set-up. All but one include a control group and three feature a more complex factorial design, in which larviciding is combined with another intervention. Before reviewing the evaluative assessments of the trials, their respective types of community engagement need some clarification.

**Table 1:** Basic features of selected community-based larviciding interventions

Country (location type) [period]	Type of intervention design [scale of LSM arm/trial]	Community engagement	Academic source(s)
#1. Tanzania (urban) [2006–2008]	Non-random trial with control [12/67 neighbourhoods]	Sensitization: No Spraying: Yes Supervision: Yes	[34, 38]
#2. Tanzania (rural) [2012–2013]	Randomised 2 × 2 factorial design; (1) LSM; (2) early detection and treatment [12/24 villages]	Sensitization: Yes Spraying: Yes Supervision: No	[29, 33]
#3. Burkina Faso (rural/semi-urban) [2013–2015]	Cluster-randomised trial with control; treatment varied by intensity (50% or 100% of sites sprayed) [85/127 villages]	Sensitization: Yes Spraying: Yes Supervision: No	[28, 36, 40, 41]
#4. Kenya and Ethiopia (rural) [2013–2015]	Cluster-randomised 2 × 2 factorial design; (1) LSM; (2) community education and mobilisation (CEM); all arms: ITN distribution [24/48 villages]	Sensitization: Yes/No (built into treatment) Spraying: Partly Supervision: No	[27]
#5. Botswana and Zimbabwe (rural) [2015]	Non-random trial with control [2/4 villages]	Sensitization: No Spraying: Yes Supervision: No	[32]
#6. Ethiopia (rural) [2016–2018]	No control group; LSM packaged with ITN + CEM [12/12 villages]	Sensitization: Yes Spraying: Yes Supervision: Partly	[30]
#7. Malawi (rural) [2016–2018]	Cluster-randomised 2 × 2 factorial design; (1) LSM; (2) structural housing improvement; all arms: community workshops [33/65 villages]	Sensitization: Yes Spraying: Yes Supervision: Yes	[31, 35, 37, 39]

It is not straightforward to position the intervention modalities unambiguously on one of the steps of Whittaker and Smith’s [24] CE-ladder, as the ‘high-effort activities’ involved in larviciding (see step 3), notably the spraying itself, is often limited to a small designated group of community members, in which case the engagement level of the community at large remains unknown. The third column of Table 1 characterizes CE per trial by distinguishing community-wide sensitization efforts, community-based larvicide spraying (implementation), and self-supervision of the overall process. A first observation is that the actual application of *Bti* is typically delegated to specific community members who are selected by experts, sometimes in consultation with community leaders, and trained by them for the tasks assigned. Apart from spraying, these field tasks occasionally include the identification of mosquito breeding sites and the sampling of larvae for entomological monitoring. The Kenya/Ethiopia case (#4) is an outlier, as half of each sprayer team consisted of outside experts alongside the ‘mosquito scouts’ from the community [27]. Individuals recruited for the spraying teams often fulfil the role of liaison with the wider community on the intervention, such as the designated Community-Owned Resource Persons (CORPs) in Tanzania (#1) or the Community Key Informants (CKIs) in Burkina Faso (#3). Efforts to sensitize the wider community on the importance of LSM often go beyond the assignation of liaison persons. In all but two cases a more comprehensive approach is built in, featuring, among others, “on-site visits of a communications officer and regular radio broadcasts” in Burkina Faso [28], “posters describing the project […] to display in a central public space” in rural Tanzania [29], and “coffee ceremony sessions and school-wide events” in Ethiopia [30]. The intervention in Malawi represents the most integrated community mobilization approach, as it is embedded in an existing community-led programme set up by a non-government organization (NGO) and series of malaria-specific community workshops were performed by ‘health animators’ as core intervention in all treatment arms [31].

The limits of community engagement are reflected most clearly in the supervision of the larviciding campaign, which often falls to outside experts. For example, the trial in the border region of Botswana and Zimbabwe made sure that “community volunteers […] worked under the full supervision of an entomologist seconded to the study by the Ministry of Health” [32] and in rural Tanzania “a trained entomology supervisor was on-site throughout the three-month intervention period in each year to […] conduct quality control spot checks” [33]. Occasionally, supervision became more hybrid, as in Ethiopia, where the ‘mosquito scouts’ who were primarily sprayers in 2013–2015 (#4) gained a more supervisory role in 2016–2018 (#6), even though the involved research institute carried the final responsibility for surveillance [30]. Two trials come close to full delegation of supervisory tasks to communities. The Urban Malaria Control Program (UMCP) in Tanzania (#1) transferred responsibility for routine mosquito control and surveillance to the CORPs [34] and in the Majete Malaria Project (MMP), Malawi, around 10–12 individuals formed a village LSM committee entrusted with the coordination of all malaria control activities at village level [35].

Most of the sources consulted to inform Table 1 aim to establish quantitative evidence on the effectiveness of the community-based trials, using a variety of objective indicators, including the density of *Anopheles* ssp. mosquitoes in different stages of the larva-to-adult lifecycle, infectious bites per person, and malaria positivity rates in rapid diagnostic tests (e.g. [36, 37]). Some assessments are unreservedly positive regarding the value added of the CE-component, such as Asale et al.’s [30] statement that “[t]he increased engagement of communities in malaria control and prevention has led to a dramatic decline in both the vector population and the disease burden” (p. 12) in Botor-Tolay district, Ethiopia. Yet, a trade-off between the delegation of *Bti* spraying to the community level on the one hand, and effectiveness on the other, was observed in rural Tanzania. Berlin Rubin et al. [33] observed a satisfactory coverage rate of community-based spraying teams (80% of the mosquito breeding habitats identified), but under-dosing of larvicide occurred in almost three out of every four sites inspected. While acknowledging incomplete adherence to protocols in the urban context of Dar es Salaam, Geissbühler et al. [38] nonetheless conclude that “larval control can have such clear benefits, even when applied sub-optimally”, which in turn “questions the assumption that very high levels of programmatic performance are essential for this approach to deliver epidemiological impact” (p. 10).

While the primary objective of these evaluation studies is to quantify the treatment effect of community transfer on entomological and clinical outcomes, several pilots also ran qualitative inquiries into process aspects, occasionally touching on experiences of ownership. The most comprehensive qualitative account emerges from the MMP intervention in Malawi’s Chikwawa district. Gowelo et al. [35] report on focus group discussions and in-depth interviews that were selectively targeted to capture variation in observed community ‘buy-in’. Individual communities were stratified for this purpose on the criterion of exhibiting either above- or below-average motivation and participation. Several barriers to participation are identified, but the most intriguing insight concerns the mixed feelings that part of the ‘ordinary’ community members harboured vis-à-vis the LSM committee members tasked with supervision, allegedly for the financial compensations they received. Furthermore, commitment to LSM is gauged from effectiveness as perceived by the informants themselves: “The community members perceived a visible decline in malaria cases in their communities, which they attributed to their work. They indicated that such achievements encouraged them to work towards more reductions in the malaria burden” (p. 7) [35]. Commitment is not explored in terms of community members’ willingness-to-pay for LSM, however. Community engagement enters the MMP’s comprehensive costing exercise as ‘societal’ cost [39], rather than as potential co-funding modality.

To our knowledge, only the trial in Burkina Faso (Kossi province) operationalized local commitment to LSM into willingness-to-pay. A survey was conducted in 36 villages located within treatment areas towards the end of the two-year larviciding intervention in 2015, in which respondents were asked to pick one out of five pre-specified amounts (CFA 100–1000 range) as their WTP. Almost two-thirds of the 446 respondents reported that they were willing to contribute at least CFA 500 (US$0.85) on an annual basis [28], which the authors qualify as “strong” in view of their prior costing exercise [40]. Such robust willingness to contribute also emerged from parallel qualitative fieldwork [41]. The authors interpret the strong motivation on the part of the community to support future LSM in reference to high levels of perceived effectiveness, rather than to ownership in the form of agency, which was limited to the act of spraying itself in the Burkina Faso case.

### From agency to commitment: a missing link in sustainable larval source management?

**Fig. 1:**
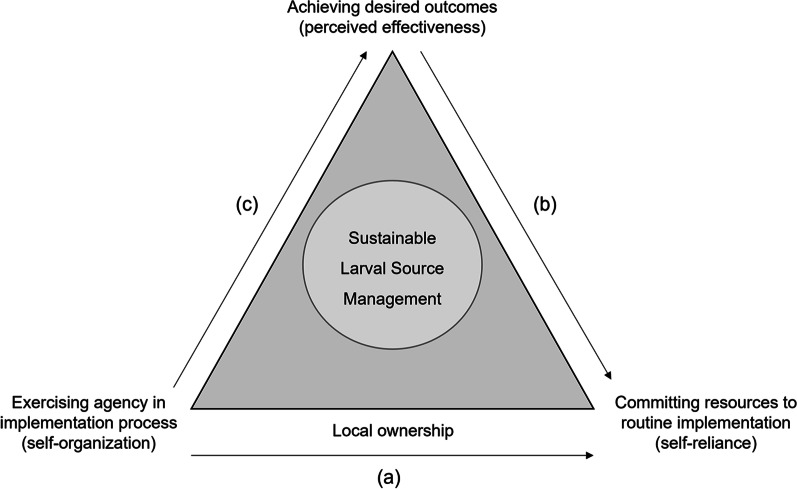
Analytical framework

This review reveals that the success of local ownership –in its agency dimension– is primarily evaluated in light of outcome effectiveness. The instrumental value that community agency in the larviciding process may exert on the commitment dimension of local ownership remains implicit. If any, an indirect link to WTP runs through perceived effectiveness. Hence, the question whether agency as such, regardless of its impact on effectiveness, is capable of fostering financial commitment remains open. It is exactly this hypothesis of more agency producing a stronger ‘buy-in’ of community resources that we put to the test. The triangle in Fig. 1 visualises our analytical framework. The direct connection between the twin dimensions of local ownership at the base of the triangle (arrow *a*) is our main focus, while existing studies either zoom in on arrow (*b*) from (perceived) effectiveness to commitment, or, more prominently, on arrow (*c*) from agency to effectiveness.

The value added of our study rests on two design aspects. First, we vary the level of agency within a single trial, where one group operates under a modality very similar to the one in Burkina Faso (#3) or the LSM-only group in rural Tanzania (#2), while the second group most closely resembles the LSM-only group in Malawi (#7). The crucial difference is that outside experts take a supervisory role in the first case, while the organization in the second case is fully community-led, including the responsibility of oversight. These two modalities arguably correspond to the third and fourth step of the CE-ladder, respectively. Please note that the experts that guided the intervention in the first group were of Rwandan nationality, so that the ‘outsider’ label refers to not being part of the community and does not imply ‘foreign’ or ‘Western’.

The second distinctive feature concerns the application of a bidding game procedure to measure WTP (see Dambach et al. [28] for a standard survey elicitation approach). Also, we compare WTP at baseline, when respondents did not yet anticipate on the (type of) larviciding trial, with an ex-post measurement taken two years after the start of the six-month trial. This timing has the advantage that the involved farmer communities were able to form some perception about how long the impact of a larviciding campaign lasted, once the spraying routine had come to an end. The control group has also been subject to repeated WTP measurement. While before-after comparisons of WTP for malaria control in Africa are not unique, such as Uzochukwu et al.’s [42] study on the willingness-to-pay for rapid diagnostic tests in eastern Nigeria, we have not come across applications concerning community-based LSM.

## Methods

### Study setting

The study was conducted in the sub-district (sector) of Ruhuha, located within Bugesera district of Rwanda’s Eastern province. Ruhuha covers 54 km^2^, ranges in elevation from 1300 to 1573 m above sea level, and hosts an estimated 26,199 inhabitants across 35 villages, which are clustered into five sub-sectors (cells). Lake Cyohoha separates it from Burundi to the south. The sub-district features four low-lying marshland areas connected to the Akagera river system, each of which is used for rice cultivation by a corresponding farmers’ cooperative. Villages closer to irrigated rice fields have been shown to host higher densities of *Anopheles gambiae* s.l., predominantly *Anopheles arabiensis* and to a lesser extent *Anopheles gambiae* s.s., resulting in higher self-reported malaria incidence in these locations [43].

Within the national context, Bugesera district featured among the 19 districts, out of a total of 30, that were classified as carrying a high malaria burden at the time of the pilot in 2015 [44]. Moreover, the intervention took place in a timeframe (2013–2016) where malaria incidence rebounded strongly nationwide, putting Rwanda off track for achieving pre-elimination status, as envisaged in the country’s Malaria Strategic Plan 2013–2018. Instead, malaria incidence increased from 48 to 403 cases per 1000 individuals between 2012 and 2016 [45]. Rwanda’s Eastern province took a more than proportional share of the burden [46], which may partly explain its relative underperformance in overall human development [47].

In terms of food security, by contrast, Eastern province outperformed the rest of the country, except Kigali city, in 2018 and Bugesera district recorded the strongest improvement in food security over the 2015–2018 period, reducing its share of food insecure households from around 30% to slightly below 10% [48]. It is plausible that the intensification of rice farming in the district was instrumental in this achievement, although the investments by international development actors to promote wetland rice cultivation in Bugesera seem to have mostly bypassed Ruhuha sub-district. Rice farming households in the study area remain small with plots in the 0.1–0.3 ha range according to records obtained from local rice cooperatives. The overall poverty headcount in Ruhuha was calculated at 34% in 2013–2014, roughly on par with the Bugesera average [49]. Ruhuha’s small urban centre, which carries the same name, featured a 21% poverty rate, implying that its rural hinterlands are more seriously afflicted by poverty (headcounts up to 50% in some villages). Ruhuha town also hosts the sub-district’s only health centre, which is supported by an outreach network of 140 community health workers, i.e., roughly four per village.

### Study design

The LSM intervention was part of the Malaria Elimination Program for Ruhuha (MEPR), a five-year, multidisciplinary, and internationally funded program in which researchers from the Netherlands and Rwanda collaborated on a range of malaria control activities in the area. Sensitization of the local population on malaria formed an integral part of the approach, such as through the organization of ‘open space’ meetings to bring out local priorities and bottom-up proposals (see [50] for outcomes of this participatory platform). Also, a network of Community Action Malaria Teams (CMATs) was established mid-2014 as liaison between health professionals and the communities in implementing malaria prevention and control interventions. Each village recruited three individuals to form its CMAT, consisting of a youth representative, a community health worker, and a village leader. In the run-up to the larviciding trial, the research team selected two smaller teams from this pool of CMAT members to carry out entomological monitoring on a set of pre-identified surveillance points across the marshlands included in the intervention. Unlike the LSM program in Botswana/Zimbabwe [32], where larval surveillance and *Bti* spraying were concurrent activities delegated to one and the same team, these were kept as distinct activities in Ruhuha and carried out by teams with minimal overlap in composition.

**Fig. 2:**
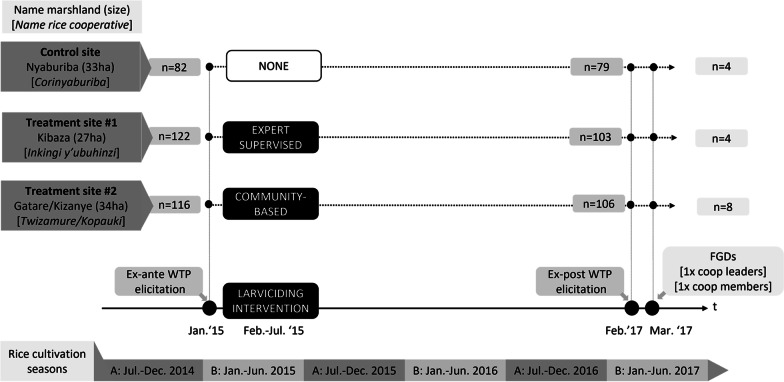
Overview of study design

Contacts with the four rice cooperatives operating in Ruhuha, together representing a membership base of 1914 rice farmers, were established to first organize a baseline assessment of attitudes towards LSM interventions, including members’ willingness to contribute to such an initiative. Respondents were informed at baseline (January 2015) about the future prospect of a pilot, but no explicit commitment nor tentative timelines of the intervention were communicated until after this round of data collection. The quasi-random allocation of cooperatives into the ‘expert supervised’ (henceforth ES) arm, the ‘community-based’ (CB) arm, and the control arm, respectively, took place after the survey, but before processing the results in order to avoid selection bias on farmers’ attitudes towards the pilot. To balance the intervention scale across the arms, the two smaller cooperatives were allocated the same treatment. The intervention sites are specified in more detail on the left-hand side of Fig. 2. Note that a minimum distance of 5 km was reserved between sites, which exceeds the observed maximum flying range of *An. gambiae* s.l. mosquitoes (1.7 km) [51], such as to prevent spill-over effects.

A first difference between the ES and CB arms concerned the recruitment of sprayer teams. In consultation with cooperative leaders, it was first agreed that sprayers should either be CMAT members or rice farmers, and, additionally, reside in a village neighbouring the areas targeted for *Bti* application. While the research team recruited sprayers through selection interviews in the ES arm, this responsibility fell to the leaders of the involved rice cooperatives in the CB arm. The research team only approved the proposed team lists upon verification that these met the pre-agreed criteria. Among the 39 community members that were eventually deployed for spraying, rice farmers outnumbered CMAT members by 3:1 approximately. A second difference between ES and CB consisted in the supervisory arrangement. Supervision of the sprayer team in ES was performed by a trained entomologist who was part of the research team (author EH) and oversaw the distribution of spray pumps and the *Bti* product, as well as the spraying routine itself. This arrangement also required the submission of daily reports to the research team office located at the health centre. On the other hand, supervision in CB was self-organized under responsibility of the cooperative leadership, who had no prior larviciding experience. Weekly reports were requested by the research team, but a strict ‘no interference’ policy was maintained. A final difference referred to logistics. Spraying materials, such as equipment and personal protective gear, were stored at the health centre in Ruhuha town in the case of ES, while these were managed on-site, more specifically in the warehouses of the rice farmer cooperatives, in the CB treatment. Note that sprayers in both treatments received minimal (and identical) financial compensation from the project for their efforts.

Larviciding started in February 2015 in both treatment arms, after all sprayers had received a five-day training on *Bti* application supported by the manufacturer. Among others, the training sessions covered the calibration of sprayer pump nozzles, the spraying speed, flow and application rates, and culminated in a field-based simulation of spraying using water. The stipulated dosage was 0.3 kg of *Bti* granules per hectare (see [52] for full technical details). *Bti* spraying was planned to be done weekly for a timespan of six months, which was strictly observed in the supervised arm. The intervention period closely overlapped with one cultivation season (season B) up to and including the start of the next one (season A), as shown at the bottom of Fig. 2. The ex-post assessment in 2017 (February/March) again took place in the B-season, 24 months (and four cultivation seasons) after the start of the intervention.

Our main interest is to test for any differential impact on WTP between the (low-agency) ES and (high-agency) CB arms, using the control group as a point of reference. Ex-ante WTP elicitation concerned 320 cooperative members randomly selected from cooperative membership records in 2015 and these same individuals were approached to participate in the follow-up WTP assessment in 2017. No replacement by spouse or other household member was accepted in this follow-up. In total a panel of 288 respondents was obtained. Thirty-two baseline respondents were not available in 2017 for a variety of reasons, including (temporal or permanent) migration, livelihood switches out of the rice sector, or demise. While this 10% overall attrition rate should not pose a serious threat to validity [53], attrition proved relatively high in the ES group (15.6%). Baseline respondents in ES unavailable for re-interview did not prove systematically different, however, from available group members in terms of WTP at baseline (*t* =  − 0.76; *p* = 0.451). A breakdown into sub-samples per treatment arm, obtained from proportional sampling on rice cooperative membership, is shown in Fig. 2 for both the baseline and the end-line measurement.

Overall, our study followed a sequential (quant-to-qual) mixed-methods design [54], since the quantitative impact assessment on the 2015–2017 panel was complemented with a qualitative inquiry at end-line (March 2017). This took the form of two focus group discussions (FGDs), one with cooperative leaders and one with ordinary members. Each of the intervention arms was represented in both FGDs (see Fig. 2 for participant numbers per cooperative). The age of participants ranged between 29 and 53 years (average of 40 years) and the gender balance was only slightly male-biased (56% male).

### Data collection

In order to obtain WTP values for a six-month larviciding campaign, we used a contingent valuation approach, which relies on stated rather than revealed preferences. It “attempts to assess the worth of a prospective intervention directly, by asking subjects to nominate their monetary valuations of the benefits perceived or expected to result, were the intervention to be made available” (p. 289) [55]. Hence, WTP statements do not carry financial consequences for respondents and, consequently, introduce the problem of ‘hypothetical bias’ [56]. It is disputed, however, whether incentive-compatible valuation methods, such as auctions or lotteries, are necessarily superior to contingent valuation methods [57]. Oerlemans et al. [58] point out that hypothetical bias in contingent valuation becomes more problematic, the wider the asymmetry in prior information about the prospective intervention among respondents. In our case, this information asymmetry is limited, as only 13% of the baseline respondents were aware of LSM using biological larvicides before it was piloted in the area [59]. More generally, WTP studies have established that the more familiar a respondent with the intervention under evaluation, the smaller the hypothetical bias [60]. This implies that we should expect less inflationary pressure on WTP due to hypothetical bias at follow-up than at baseline, at least in the two treatment arms, following exposure.

Within the contingent valuation approach, we preferred a bidding game format over alternative formats such as open-ended, payment card or dichotomous choice formats. The bidding or bargaining format starts out with a WTP proposed by the researcher that a respondent accepts or rejects, and depending on the decision, a respondent is bid up and down by the interviewer until reaching maximum WTP, requiring only “yes or no” responses to each bid. Its prime advantage is the break-down of the choice process into a series of simplified (dichotomous) steps, thus offering guidance to respondents in their decision-making, which in turn facilitates careful consideration [61]. This is an important merit over alternative formats in light of the education profile of our sample; one in four has not attended school or did not complete primary education. Bidding comes at a disadvantage of potential respondent fatigue following multiple decisions, which we minimized by restricting the number of iterations to five after the starting bid. Bidding also triggers “anchoring bias” (p. 52) [61] if the starting bid is inadvertently taken as a cue for actual value. To gauge the magnitude of such an anchoring bias, participants were randomly assigned to either a low or high starting bid (1500 or 2500 RWF, respectively). Analysis of the WTP values obtained at baseline indeed bears out a significant starting-bid effect [62]. However, since starting bids in the ex-post elicitation were intra-individually synchronized with the algorithm used at baseline and given the focus on WTP *changes* over 2015–2017 rather than on absolute WTP levels, starting-bid effects should be effectively neutralised.

Along with the WTP elicitations, respondents were asked a set of additional survey questions during face-to-face interviews. Both were administered under field supervision of the first author (AR) by a team of ten experienced, Kinyarwanda-speaking surveyors. They made use of Open Data Kit Collect setup, installed on tablet devices, to record responses in digital form and upload these via an on-site server. Both the bidding procedure and the questionnaire were pre-tested to check for proper understanding of the terminology used and to reformulate questions open to ambiguous interpretation. Written informed consent was obtained from all respondents prior to an interview. The follow-up questionnaire in 2017 overlapped strongly with the baseline questionnaire, and most questions on background characteristics were retained as a reliability check. However, a module was added that contained questions on how the process and impact of larviciding had been experienced, administered to the ES and CB arms only. Given the focus on ownership, respondents in all three arms were asked the more general question which actors they believe should ‘own’ the malaria problem in their locality (not included in baseline).

During the focus group discussions, moderated by the first and second author (AR and LK), participants were invited to voice their experiences in a more open format, based on a loosely structured discussion guide. Discussions covered the spraying process itself, its perceived impact in terms of mosquito presence and corresponding malaria risk, and the role of different actors in taking responsibility for malaria control. Rules of engagement were mutually agreed at the start of each conversation. Special care was taken to involve control group representatives into the discussions, despite their lack of first-hand experience on LSM. The FGDs, for which no incentives were provided except for snacks and drinks, lasted between 1 and 1.5 h.

### Data analysis

WTP change was quantitatively assessed using a difference-in-difference (DID) approach to compare intervention arms (pairwise), eliminating time-constant unobservables [63]. Since treatment allocation was not randomised at the individual level, the DID estimates are conditioned on matching propensity scores across arms [64]. First, propensity scores were generated from a probit regression on group membership, using a set of background variables at baseline, indicating an individual’s ex-ante likelihood of placement in a specific arm. Subsequently, DID estimates were computed using the “diff” command in STATA version 17, using kernel matching as propensity score matching technique. Kernel matching is a non-parametric technique that compares each treated unit to all units in the control group, but the latter are weighted with the inverse of the distance in propensity score between treated and control unit [64]. Hence, the reference group for a particular respondent is constructed such that the most similar individuals carry disproportionate weight. We report DID estimates both over the full range of propensity scores as well as on common support only. The latter ignores the most dissimilar observations.

Finally, the qualitative analysis was based on full transcriptions of the FGDs, which were subjected to an open coding process to triangulate and complement the quantitative information obtained from the survey.

## Results

This section first reports on how WTP for larviciding changed over the two-year timeframe across the treatment arms and, subsequently, explores to what extent the farmers’ mindset regarding community ownership and their perceptions of LSM effectiveness can explain the observed dynamics in financial commitment.

### WTP change

**Fig. 3:**
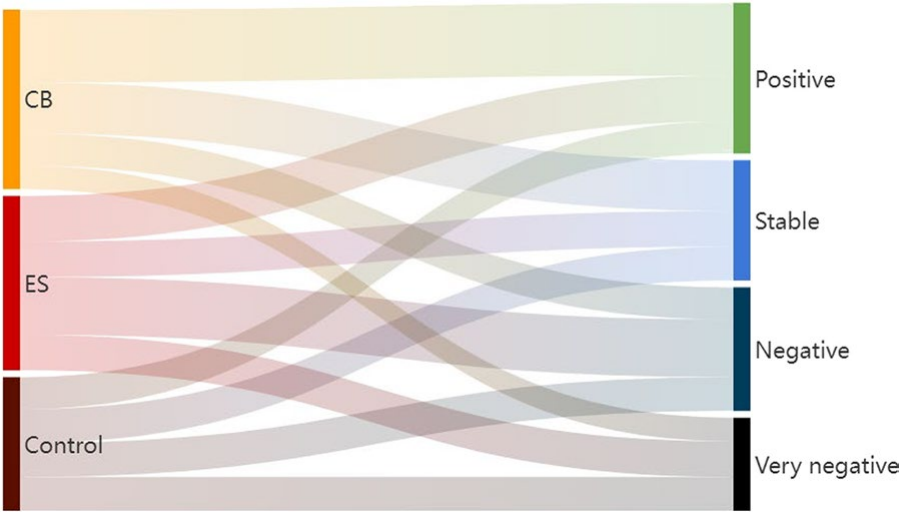
Sankey diagram of WTP change, by treatment group

Across the entire panel (*n* = 288) the average respondent’s pre-intervention WTP was 1548 RWF in 2015, while this dropped to 1318 RWF in the 2017 follow-up. If we consider small differences (< 250 RWF) between the 2015 and 2017 bids for a given respondent as ‘stable’, it emerges that reducing WTP was the norm in both the ES group and the control group. For every control farmer who increases WTP, there are roughly two who decrease WTP, and this ratio is almost identical for ES farmers. Among CB farmers, on the other hand, the opposite tendency can be observed; increases in WTP outnumber decreases by a 4:3 ratio. Overall, mean WTP values fall sharply in the control and ES arms, while increasing slightly in the CB group. Table 2 provides a more detailed distribution of responses per arm, distinguishing between ‘moderate’ and ‘large’ changes (cut-off at 1000 RWF). Figure 3 visualizes the same, but lumps moderate and large WTP increases into a single category (‘positive’) for parsimony. WTP changes in the positive direction are clearly most closely associated with the CB group.

**Table 2:** Relative distribution (%) of direction and degree of change in *ex-post* WTP (2017) compared to *ex-ante* WTP (2015), by treatment status

Change category (*x* = WTP_2017_ − WTP_2015_ in RWF)	Control (*n* = 79)	ES (*n* = 103)	CB (*n* = 106)
Large increase (*x* > 1000)	12.7	9.7	17.0
Moderate increase (250 < *x* ≤ 1000)	11.4	16.5	23.6
Stable (− 250 ≤ *x* ≤ 250)	25.3	20.4	28.3
Moderate reduction (− 1000 ≤ *x* < − 250)	25.3	33.0	17.9
Large reduction (*x* < − 1000)	25.3	20.4	13.2
	100.0	100.0	100.0

However, we need to check for bias resulting from systematic pre-intervention differences between the treatment clusters. For this purpose, propensity scores are generated from a set of 15 baseline variables, as shown in Table 3. Group means are presented for each matching variable in columns 1–3 and the probit coefficients on group membership in columns 4–6. This reveals that the composition of the groups is unbalanced in a number of aspects. Among the most salient is land size, which is largest in the control arm and smallest in ES. As far as malaria is concerned, the control group stands out for a higher perceived frequency of mosquito biting, although this does not translate into a higher perceived malaria burden. Interestingly, the three groups similarly acknowledge that irrigated rice cultivation serves as a malaria hotbed and also concur in their expectation that *Bti* will prove effective in reducing malaria risk. However, disparities related to LSM also emerge. The control group shows significantly weaker trust in the safety of *Bti* for humans and other living organisms. ES, on the other hand, is an outlier in terms of awareness of larviciding as a malaria control tool; one third has heard about it before, against marginal shares in the CB and control groups. Finally, systematic differences in a number of demographic variables emerge, as well as in the importance of rice in a household’s livelihood. On average, rice accounts for slightly over 60% of household income among controls, while remaining below this mark in both treated groups.

**Table 3:** Propensity score variables (2015): mean value by treatment group (columns 1–3) and probit coefficients for group membership (columns 4–6)

	(1) Control	(2) ES	(3) CB	(4) ES (vs. Control)	(5) CB (vs. Control)	(6) CB (vs. ES)
High starting bid (2500 instead of 1500 RWF)	0.47	0.49	0.54	− 0.15	0.46***	0.05
Age of respondent (in yrs)	46.5	44.0	43.7	− 0.01	− 0.02***	− 0.01
Gender of respondent (male = 1)	0.57	0.56	0.54	0.16	− 0.35**	− 0.34**
No primary schooling (or not completed)	0.29	0.18	0.26	− 0.49*	0.06	0.28
Number of household members	5.7	6.0	5.4	0.02	− 0.10**	− 0.13***
Number of under-five children	0.5	0.8	0.8	0.19	0.37***	0.04
Number of years in cooperative	7.9	7.7	8.0	− 0.07*	0.07**	0.02
Share of rice income in total income	62.6	58.9	56.8	0.00	− 0.01**	− 0.01**
Size of land (in *are* = 100m^2^)	7.9	3.7	5.9	− 0.35***	− 0.10***	0.25***
Perceived importance of rice cultivation for malaria risk (1 = not at all important, …, 4 = very important)	3.5	3.1	3.4	0.00	− 0.02	0.24*
Frequency of mosquito bites in rice fields (1 = almost never, …, 4 = very often)	3.8	3.4	3.4	− 0.71***	− 0.49***	− 0.02
Malaria case in household in past year (0 = no; 1 = yes)	0.68	0.65	0.77	0.06	0.68***	0.22
Ever heard of larviciding (0 = no; 1 = yes)	0.01	0.33	0.04	2.36***	0.56	− 1.74***
Confidence in larviciding to effectively reduce malaria risk (1 = not at all, …, 4 = very much)	3.4	3.4	3.5	− 0.11	− 0.12	0.00
Trust in safety of larvicide application (1 = not at all, …, 4 = very much)	2.8	3.2	3.2	0.65***	0.80***	0.08

***, **, *Significance at 1%, 5% and 10%, respectively

**Fig. 4:**
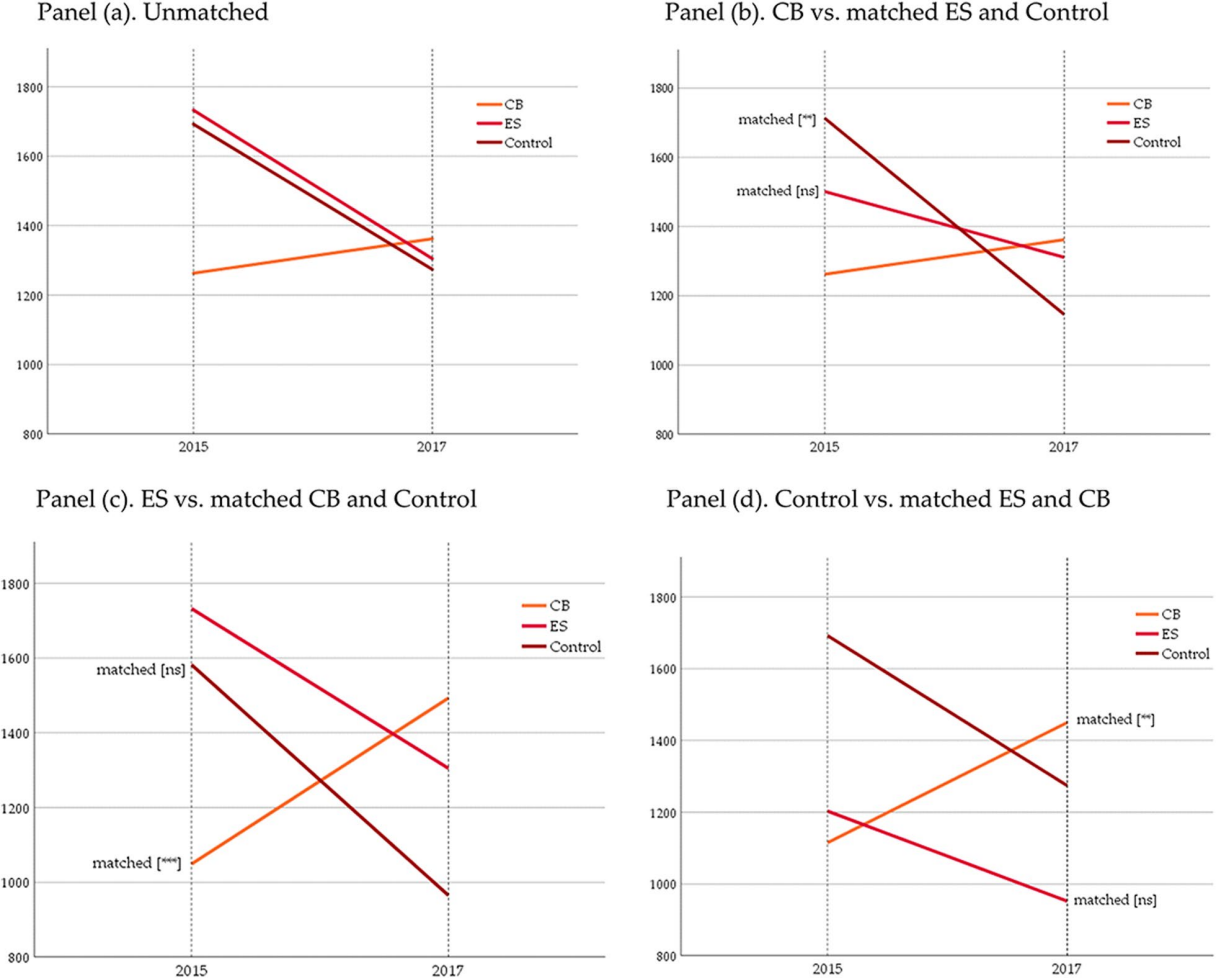
Mean WTP change, by group; unmatched comparison (panel **a**) and each group plotted against matched comparisons (panels **b**–**d**). [ns, not significant]

A comparison of the groups after matching offers an important nuance to the observed WTP dynamics prior to balancing on baseline characteristics. The picture that arises from unmatched DID analysis, as shown in panel (a) of Fig. 4, suggests that WTP reductions in the control and ES groups are almost identical. However, all three matching scenarios (panels b-d), reveal a steeper slope for the control group, implying a sharper reduction, compared to ES. Panel (b), in which CB acts as the benchmark and is compared to matched versions of ES and control, illustrates this clearly. The ES arm takes an intermediary position between control and CB and the difference-in-difference analysis (see Table 4) even fails to detect a significant difference between ES and CB behaviour. At the same time, if we consider ES as reference (panel c), a significant reduction is revealed compared to its matched comparison in CB. Mean WTP drops by 870 RWF (or 847 RWF on common support only). Overall, the core result still holds that WTP in CB moved in the opposite direction from that in the control and ES arms, but ES is apparently less on par with the control group than first inspection suggests, as the magnitude of reduction is smaller in the expert-supervised group than in the non-sprayed group.

**Table 4:** Pairwise diff-in-diff results on WTP change after Kernel propensity score matching; results on common support in italics

Reference group	Versus CB match	Versus ES match	Versus control match
CB		+ 290 [t = 1.28] + *268 [t* = *1.22]*	+ 667 [*t* = 2.44**] + *726 [t* = 2.49**]
ES	− 870 [*t* = 3.84***] *− 847 [t* = 3.42***]		+ 189 [*t* = 0.81] + *220 [t* = *0.84]*
Control	− 752 [*t* = 2.48**] *− 760 [t* = 2.43**]	− 167 [*t* = 0.68] *− 184 [t* = *0.68]*	

***, **Significance at 1% and 5%, respectively

Despite the nuance that balancing the groups brings in, the results appear consistent with a narrative in which the stronger autonomy of the larviciding campaign in CB fosters local ownership of malaria control efforts, and thereby sustains willingness-to-pay for future spraying interventions, whereas expert-led interventions undermine intrinsic motivation by fostering a mindset of dependency. The WTP fall in ES would thus mimic, at least to some extent, the loss of commitment in the non-intervention group. However, in order to match this narrative to our case, indications of enhanced ownership in CB and/or eroded ownership in ES should be observed in support.

### Local ownership

Respondents were asked in the post-intervention survey to indicate which actors carry responsibility with respect to local malaria control in their opinion. Each respondent scored the responsibility of the following actors on a ten-point scale: (a) national government, (b) local government, (c) international donors, (d) local people themselves, and (e) the community of rice farmers. For each respondent these scores were converted into a ranking of actors according to the weight of responsibility awarded (rank 1 for most responsible and 5 for least responsible actor). Ties were awarded *ex aequo* rank orders. Table 5 reports on the mean rank per actor for each intervention arm.

**Table 5:** Mean rank of actor-specific responsibility awarded by treatment group (rank order in parentheses)

Actor	Control	ES	CB
Government	1.27 (#1)	1.16 (#1)	1.23 (#1)
International donors	1.76 (#2)	2.75 (#2)	2.61 (#2)
Local authorities	2.81 (#3)	2.85 (#4)	2.81 (#3)
Local farmers	3.00 (#4)	2.77 (#3)	2.87 (#4)
The people themselves	3.90 (#5)	3.33 (#5)	3.25 (#5)

**Fig. 5:**
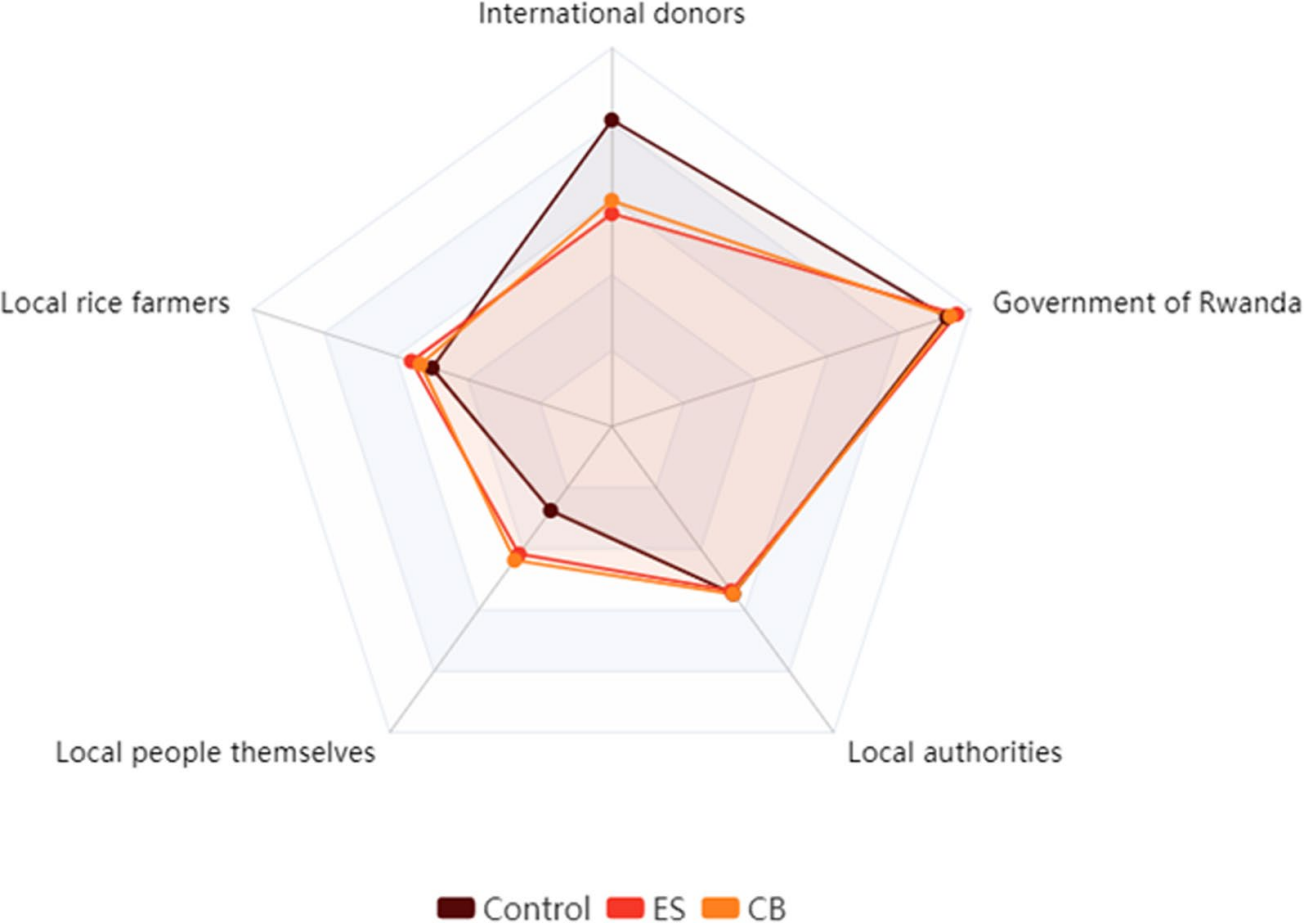
Actors’ responsibility for malaria control; ranks represent group means and assigned responsibility increases with distance

The overall rank order appears very similar across arms; only the categories of ‘local authorities’ and ‘local farmers’ switch rank in ES compared to the other two arms. Nevertheless, a striking difference arises between the control group on the one hand, and ES and CB on the other, where it concerns the responsibility of international donors versus that of the local population. This can be appreciated in Fig. 5, in which the rankings are inverted for presentational purposes; the further out along the rays in the radar chart, the higher the assigned responsibility to the respective actor. While the main responsibility lies with the Rwandan government in each of the groups, the control group puts international donors almost on par, whereas ES and CB leave a substantial gap between these two actors. This represents first and foremost a trade-off with the responsibility carried by the local people themselves, and to a lesser extent with that of local farmers. Since both ES and CB put less responsibility on international donors and more on local (non-governmental) actors, local ownership appears stronger in both intervention sites, regardless of whether experts or farmers themselves were coordinating the intervention. Note that this claim rests on the assumption that the three groups did not systematically differ on pre-intervention ‘sense of ownership’, which we were unable to verify for a lack of baseline scores on actor responsibility, but our qualitative evidence also points at strong community identification with malaria control in both treatment groups.

The participants representing ES during the post-intervention FGDs did not reflect or refer to a dependency mentality and, like their CB companions, advocated communities taking responsibility instead. For instance, upon the moderator’s question whose action is key in malaria control, ES and CB representatives concurred during the FGD with cooperative members:

*ES member*: The first person who is responsible for malaria prevention and control is a citizen; malaria can never be eliminated unless citizens themselves play active roles in combating it.

*CB member*: I agree on the view of citizens being at the forefront of fighting malaria. For e.g. concerning contributions to support *Bti* cost; I feel that if all citizens would understand the benefit and contribute, whatever they can afford, malaria can be eliminated. … Though other sectors may come in to play their roles; but citizens who are the beneficiaries should first feel responsible for malaria control; then others would come in as supplementary.

The ES participants seemed well-aware of the financial implications of such a position, as witnessed by one of the cooperative leader’s proposal during the FGD to set up a *Bti* co-funding scheme:

I feel, just as we pay taxes, contributions for fertilizers, and other co-operative fees from rice sales, we should also fill a fund for supporting *Bti* cost by deducting from rice sales after every harvest season … and the amount to be contributed by each member would depend on the price of rice for that season.

However, cooperative leaders in both ES and CB are explicitly calling on other sectors to foot part of the bill:

*CB leader*: As rice farmers’ co-operatives we could play our role and contribute towards its cost. Nevertheless, since mosquitoes have no boundary, meaning that they do not bite only rice farmers, we feel the whole community should be involved in contributing towards *Bti* cost, as well as the Ministry of Health and other government organs. All concerned sectors should discuss to find out which role each should play in supporting a *Bti* intervention.

*ES leader:* I very much agree on that issue … combating mosquitoes should involve all community members, not only rice farmers.

While ES and CB representatives sounded very similar opinions that resonated some degree of collective empowerment, the contributions of several informants from the control group befitted a more dependent attitude. They tended to voice frustration over their non-intervention status:

*Control leader*: In fact we feel we were left out, we don't know why. … So we feel, if another opportunity is there, we should be given first priority. … Since our marshland has never been sprayed, what hope do we have? Is there any chance that we shall be considered?”

This hints at a potential explanation for the observed drop in WTP in the control arm. Signalling a lack of resources in the group would raise the urgency (and deservingness) of their inclusion in a future round of spraying. Oerlemans et al. [58] acknowledge the possibility of such a “strategic bias effect” in contingent valuation, where WTP is suppressed in a bid to draw in external funding. However, this leaves the question why ES reduced WTP as well, given that there is no indication that the expert-led intervention fed a dependency mindset. The (limited) data on the actual effectiveness of the *Bti* intervention on mosquito and malaria control provide some mixed clues.

### Effectiveness

Entomological monitoring was carried out by the research team at baseline (one month prior to the start of the intervention) as well as for nine consecutive rounds with two-week intervals during the intervention period in each of the three arms, until the end of the rice cultivation cycle (July 2015). The analysis of these data, which is documented in a companion paper [52], reveals that effectiveness in terms of average *Anopheles* ssp. larvae reduction per survey round was significantly higher in ES than in CB. Since larval populations exhibit a natural tendency to decline over the course of the rice cultivation cycle due to the crowding out of breeding space for mosquitoes by growing rice plants, we observed a 22% reduction per round in the control arm, which serves as a reference point for larviciding success. The CB arm registered a 28% reduction on average per round and thus outperformed the control arm, but falling short of the reduction recorded in the ES arm, which amounted to 49%. However, it should be noted that this underperformance in CB vis-à-vis ES is chiefly attributable to a relatively low reduction rate in the first half of the intervention period, especially the first month. This is illustrated most clearly when zooming in on the number of sampled sites that contained mosquitoes in the pupae stage. The performance of CB and ES in terms of pupal occupancy rate converges in the second half, where habitat occupancy rates are effectively driven down to zero, whereas in the control arm these remain variable, and occasionally high, until the last round [52].

In case the slow onset of effectiveness observed in CB is inherent to community self-organization, the monitoring data suggest that LSM application of larval source management by well-trained professionals had advantages over implementation by rice farming communities themselves, implying a trade-off between community agency and effectiveness. This aligns with the under-dosage problem identified during community-based larviciding in rural Tanzania [33], but is at odds with Chaki et al.’s [65] finding in urban Tanzania that CORPs who were recruited through the program administrative staff performed rather poorly when compared to those who were selected at the community level, specifically in relation to the task of mosquito habitat coverage. It should be pointed out, however, that our outcome effectiveness analysis did not extend beyond the intervention period, so we are unable to check the precise window of effectiveness in both groups in an objective sense, nor did we track to what extent lower larval and pupal densities translated into lower rates of entomological inoculation or malaria positivity. To fill in this information gap, *perceptions* on effectiveness were solicited in the post-intervention survey and the focus groups, despite the potential risk of perceptual data being sensitive to bias. Sources of bias include the retrospective nature of the effectiveness question (recall bias), but, more importantly, the differential effort spent on the intervention itself. The CB group’s deeper involvement might trigger confirmation or evaluation bias, which means respondents would be tempted to disregard cues of ineffectiveness, or weigh up positive and negative cues differently, when asked to evaluate the intervention’s impact [66]. On first impression, perceptions do not diverge, however, as the FGD participants from both ES and CB invariably recounted strong impacts from the intervention:

*CB member*: Before *Bti* spraying there were many mosquitoes in rice fields; whenever we went there for weeding and carrying out other farming activities, we could see plenty of mosquito larvae and adult mosquitoes flying from rice plants; often they could bite us while working in the fields. As a result, we could get attack of malaria at least twice a month; but after *Bti* spraying mosquito larvae were destroyed and so adult mosquitoes reduced, and thus malaria cases also reduced.

*ES member*: I also have testimony to give; in my family, two children had severe malaria attacks last months. Since Ruhuha health centre failed to treat them, they were transferred to “Nyamata” hospital, where they were admitted for a week; after they were discharged, my wife also fell sick with malaria, but was treated at the health centre. Whereas during the period *Bti* was sprayed and some months later nobody in my family got malaria attack. Secondly, I happen to be a community health worker in my village; I can testify that during *Bti* spraying period, we could get very few cases of malaria; for instance, only 1 out of 20 patients we received per day had malaria. But some months after stopping *Bti* spraying, malaria cases increased, whereby more than half of the cases we received had malaria.

**Fig. 6:**
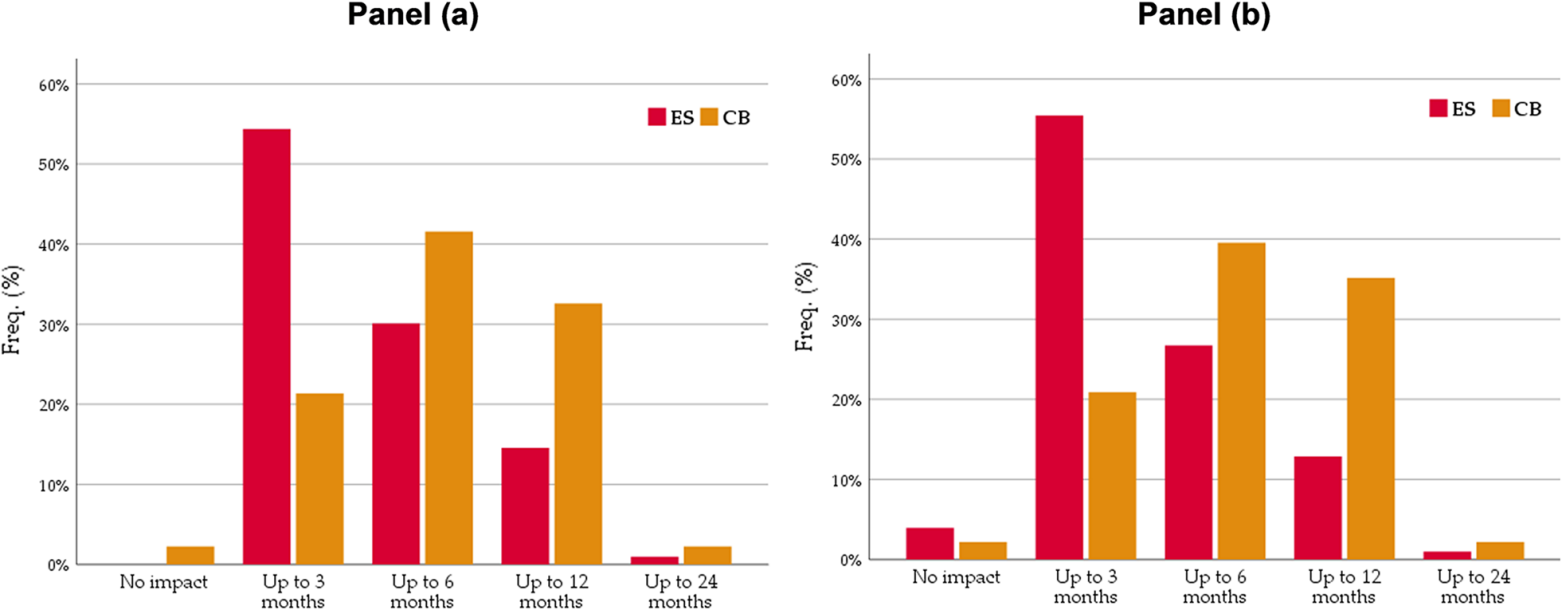
ES versus CB: relative distribution (%) of perceived duration of *Bti* impact on mosquito density in rice fields (panel **a**) and homesteads (panel **b**)

Also in the ex-post survey ES and CB respondents nearly universally confirmed positive effects from larviciding, in terms of reduced mosquito density (both in the rice fields and in their homesteads) as well as in terms of malaria risk reduction. However, the final remark in the previous quote from an ES member hints on an aspect that proves relevant in explaining WTP, i.e., the timing and magnitude of the rebound of mosquitoes and malaria infection after the LSM intervention. Responses from ES and CB informants markedly diverge. Panels (a) and (b) in Fig. 6 show the distribution of perceived impact duration in terms of reduced mosquito presence in rice fields and homesteads, respectively. Both panels convey the same message that impact persisted longer in CB than ES. The modal response in the CB group suggests that impact lasted up to six months after the spraying concluded, as opposed to only three months in ES. On average, the indicated duration is significantly higher in CB (*t* = 4.2 for rice fields and *t* = 5.2 for homesteads; in both cases *p* < 0.01).

On recall, the perceptual data thus suggest a quicker –and potentially more massive– rebound of mosquitoes in ES compared to CB. We cannot objectively corroborate to what extent, nor why, mosquitoes returned to the ES site sooner. A tentative explanation is that the impact of the CB intervention outlasted the ES intervention due to the slow onset of the former, as witnessed in the entomological data, which would signal a-synchronicity in the window of effectiveness between the groups rather than a shorter impact duration in ES per se. Notwithstanding the exact cause, this may have motivated respondents in the expert-supervised arm to adjust their ex-post WTP statements downwards, especially if the typical baseline expectation regarding the duration of the impact was closer to six than to three months. If impact proved more short-lived than anticipated, spraying campaigns need to be repeated more frequently, producing a lower WTP per six-month campaign if individual budget constraints for malaria control are relatively fixed. For most CB members, on the other hand, perceived effectiveness might have corresponded better to, or even exceeded, prior expectations, especially if they overlooked the slow onset of impact in the early days of the intervention. The difference in speed of onset between ES and CB also opens up the possibility that a rebound was more salient for ES members in comparison to CB members facing a more gradual and less pronounced mosquito density curve.

## Conclusions

The main question that motivated this study is whether the agency (self-organization) and commitment (self-reliance) dimensions of local ownership interact. More specifically, the set-up of our LSM pilot in Rwanda tested the hypothesis that a higher degree of agency results in stronger (resource) commitment at the community level. This is often implicitly assumed, as witnessed by the sequencing of efforts to build agency and to mobilize resources, respectively. The localisation of organizational responsibilities is typically prioritised, while that of financial responsibilities is backloaded, despite broad acknowledgement that both are essential for sustainable development.

At face value, the larviciding intervention in Ruhuha appears to confirm the idea that agency is a precondition for financial commitment, as WTP slightly increased in the intervention arm where rice farmer communities self-organized the campaign, while it fell markedly in the arm under expert supervision. Hence, the pernicious effect of dependency on outsiders seems at work, but this interpretation appears misguided upon deeper probing. We fail to find evidence, neither quantitative nor qualitative, that the expert-supervised intervention eroded the community’s sense of ownership over LSM as compared to the community-based modality. The more likely scenario is that the ES and CB groups behaved differently in the ex-post WTP elicitation in response to the diverging perceptions on the pilot’s effectiveness.

Revisiting the analytical framework in Fig. 1, our results suggest that the link from effectiveness to resource commitment (arrow b) trumps the direct link from agency to financial commitment (arrow a). This finding resonates well with a Rwandan proverb that one of the focus group participants brought up during the conversation: “*Agahimbaza musyi kava mu ngasire*”, which translates as “people are motivated from their achievement”. This does not imply that commitment levels are insensitive to the degree of agency, but that the effect of agency is primarily an indirect one, mediated by its effect on effectiveness (arrow c). Unfortunately, we are unable to make strong claims on whether enhanced agency helped or hurt effectiveness in the context of our intervention. While the entomological data point to suboptimal self-organized LSM implementation, respondents’ perceptions did not bear this out. The community-based arm witnessed a longer-lasting impact than the expert-supervised one. Differential timing of the larval monitoring and the post-intervention interviews may be part of the explanation for the observed dissonance.

Yet, we feel our results are sufficiently robust to question the idea that (strong) agency is a necessary condition for resource commitment to LSM. If corroborated for communities other than cooperative farmer groups, this would open up opportunities to tap into local resources whilst building local agency, rather than await the time-consuming process of building high levels of self-organization first. Moderate levels of community engagement, say, the third rather than the fourth step on Whittaker and Smith’s [24] participation ladder, may suffice. The lesson for international donors and (sub)national governments who wish to embrace the localisation agenda in the area of malaria control is therefore that it is prudent to try to cultivate the twin dimensions of local ownership simultaneously rather than consecutively.

## Data Availability

All data, transcripts, and supporting documents for this study are stored at the Data Repository of Radboud University, which can be made available upon request to the corresponding author.

## Abbreviations

*Anopheles* ssp.: *Anopheles* Subspecies
*Anopheles gambiae* s.l.: *Anopheles gambiae* Sensu lato
*Anopheles gambiae* s.s.: *Anopheles gambiae* Sensu stricto
Bti: Bacillus thuringiensis var. israelensis
CB: Community-Based
CE: Community Engagement
CEM: Community Education and Mobilisation
CKI: Community Key Informants
CMAT: Community Action Malaria Team
CORP: Community-Owned Resource Persons
DID: Difference-In-Difference
ES: Expert Supervised
FGD: Focus Group Discussions
IRS: Indoor Residual Spraying
ITN: Insecticide-Treated Net
LSM: Larval Source Management
MEPR: Malaria Elimination Program for Ruhuha
MMP: Majete Malaria Project
NGO: Non-Governmental Organization
NRDS: National Rice Development Strategy
SDG: Sustainable Development Goal
UMCP: Urban Malaria Control Program
WTP: Willingness-To-Pay
